# Hypothalamic-Pituitary-Adrenal Suppression and Iatrogenic Cushing's Syndrome as a Complication of Epidural Steroid Injections

**DOI:** 10.1155/2013/617042

**Published:** 2013-08-07

**Authors:** Joyce Leary, Arthur Swislocki

**Affiliations:** ^1^Medical Service, VANCHCS, Martinez, CA 94553, USA; ^2^Division of Endocrinology and Metabolism, Department of Internal Medicine, UC Davis School of Medicine, Sacramento, CA 95817, USA

## Abstract

Epidural steroid injections are well accepted as a treatment for radicular back pain in appropriate candidates. While overall incidence of systemic side effects has not been well established, at least five biochemically proven cases of iatrogenic Cushing's Syndrome have been reported as complications of epidural steroid treatment. We present an additional case of iatrogenic Cushing's Syndrome and adrenal suppression in a middle-aged woman who received three epidural steroid injections over a four-month period. We review this case in the context of previous cases and discuss diagnostic and management issues.

## 1. Introduction

Epidural injections of corticosteroids are an accepted and widely used treatment for radicular low back pain. Nearly 50 million in Medicare dollars went to this treatment in 1999 [1], and from 1994 to 2001, there was a 271% increase in Medicare funded lumbar epidural steroid injections [2]. A typical epidural steroid is triamcinolone in 40 mg or 80 mg doses. Some expert recommendations promote giving up to 3 injections within a year with a minimum of 30 days between injections. Further or more frequent injections are not recommended due to concerns about hypothalamic-pituitary-adrenal suppression [3]. Other practice guidelines provide no recommendations on maximum injection number or dosing interval [4]. The American Society of Interventional Pain Physicians-Interventional Pain Management comments in their 2009 guidelines that there is no “basis for reported assumptions and limitations” on epidural steroid injection doses or frequency and furthermore, that “administration must be based solely on patients' response, safety profile of the drug, experience of the patient, and pharmacologic and chemical properties such as duration of action and suppression of adrenals [5].” However, they go on to recommend epidural steroid injections be given no more frequently than every 1-2 weeks during the diagnostic phase and thereafter no more frequently than every 2 months.

## 2. Case

The patient is a 53-year-old Caucasian woman of Northern European ancestry referred to Endocrinology by a specialty Pain Clinic for concerns of possible hypercortisolism. She had been seen by the pain specialist for chronic radicular back pain and was given 3 doses of fluoroscopically guided epidural triamcinolone 80 mg via the caudal approach over the course of 4 months. Four weeks elapsed between her first and second doses, 10 weeks between her second and third, her last dose having been given 7 weeks prior to our initial evaluation. She noticed bloating and a buffalo hump between her second and third doses. She then noted progressive muscle weakness, difficulty rising from sitting to standing, worsening in her baseline insomnia, loss of hair on her head, redness of her chest, and acne. Furthermore, she stated her face had rounded and her arms and legs looked thin while her abdomen seemed to be expanding. She denied easy bruising, newly visible striae, increased tan or scar pigmentation, or other skin changes, but complained of increased hair growth on her face including thickening of her sideburns. While there was a family history of hirsutism, there was no family history of endocrinopathies. Her past medical history included chronic back pain, three uncomplicated pregnancies, regular menses until menopause at the age of 45, and long-standing hypertension. Notably, she had been told that she had “borderline diabetes” within the six months prior to her presentation. Medications included naproxen sodium, gabapentin, nortriptyline, simvastatin, hydrochlorothiazide, benazepril, calcium carbonate, multivitamin, and omega-3 fish oil.

On exam, she was obese (103.8 kg, BMI 35.9) and hypertensive (175/88 mm Hg). She was plethoric along the neck, face, and upper back. She had increased vellus facial hair. There was fat accumulation at the base of the neck. There were no striae or bruises.

Given the rapid onset of her symptoms and the complaint of hirsutism, she was evaluated for endogenous hypercortisolism and nonclassic congenital adrenal hyperplasia. A random afternoon cortisol level drawn three weeks after her last epidural injection (four weeks prior to our initial evaluation) was 16.6 nmol/L (0.6 mcg/dL (normal morning value 118.6–618 nmol/L) (4.3–22.4 mcg/dL)), and repeat at 10 am four weeks later was 63.5 nmol/L (2.3 mcg/dL). Bioavailable testosterone level was low at 0.045 nmol/L (1.3 ng/dL (normal: 0.052–0.326 nmol/L) (1.5–1.9 ng/dL)), and DHEA-S level was undetectable. A midnight salivary cortisol level three weeks after our initial visit was only 0.77 nmol/L (0.028 mcg/dL) (normal < 3.09 nmol/L (0.112 mcg/dL)). Prolactin level and thyroid function tests were normal. Chemistries were consistently normal without aberration in potassium, sodium, or creatinine. Laboratory data pertinent to our initial evaluation are summarized in Table 1. 

Although no adrenocorticotropic hormone (ACTH) level was obtained, laboratory investigations were felt to be consistent with hypothalamic-pituitary-adrenal (HPA) suppression from exogenous steroids. The patient was empirically placed on prednisone 5 mg daily, was given instructions on stress-dosing of steroids, and was provided with a dose of injectable hydrocortisone to be used in case of emergency. At one and two months after our initial visit (2-3 months after her last epidural injection), the prednisone was held for 24 hours, and repeat morning cortisol levels remained low at 80.0 and 63.5 nmol/L (2.9 and 2.3 mcg/dL), respectively (Table 1). The patient underwent a cosyntropin stimulation test four months after her last epidural injection, which showed a suboptimal rise from 209.7 to 389 nmol/L at 60 minutes (7.6 to 14.1 mcg/dL). A random 17-hydroxyprogesterone was not elevated at 0.21 nmol/L (7 ng/dL). Shortly thereafter, the patient underwent an outpatient rotator cuff repair. She was instructed to increase her prednisone to 15 mg the day of her surgery and to return to 5 mg daily afterwards. Her surgery and postoperative period were uneventful.

Given her morning cortisol level remained suppressed, the patient's prednisone dose was tapered slightly to 4 mg daily. One month later (six months after her last epidural injection), her cortisol level was normal at 328.3 nmol/L (11.9 mcg/dL) at 0935. The same day, she also demonstrated an appropriate response to cosyntropin with cortisol levels rising to 485.6 and 554.6 nmol/L (17.6 and 20.1 mcg/dL) at 30 and 60 minutes after stimulation, respectively. During this cosyntropin stimulation test, 17-hydroxyprogesterone rose from a baseline level of 0.73 nmol/L (24 ng/dL) to 2.3 nmol/L (76 ng/dL) at 60 minutes after stimulation, excluding 21-hydroxylase deficiency. Prednisone was then discontinued.

## 3. Discussion

Case reports and some experimental data suggest that adrenal disorders can result from epidural steroid injection. Patients can develop isolated HPA suppression, or HPA axis suppression can be accompanied by overt iatrogenic Cushing's. The potential for HPA suppression has been clearly documented and is referenced briefly by expert recommendations [3]. Meanwhile, the potential for accompanying iatrogenic Cushing's Syndrome is less well recognized and goes unmentioned in any guidelines. Moreover, Jacobs et al. assert that the lack of a detectable plasma methylprednisolone level after epidural injection in 12 patients indicated that systemic absorption does not occur, and therefore, patients are unlikely to have systemic side effects [6]. Previously published case reports of patients presenting with systemic symptoms, including the present case, do not support this assertion. In addition to the present case, five other case reports of biochemically proven adrenal suppression in patients with Cushing's Syndrome have been reported [7–11], and another four cases of patients with Cushing's features were reported in 1980 by Knight and Burnell, but no biochemical data was provided [12]. Case reports by Stambough et al. [7] and Horani and Silverberg [8] describe patients presenting with moon facies, supraclavicular fat pad, buffalo hump, bruising, peripheral edema, abdominal striae, and hypertension. In addition to similar common manifestations of hypercortisolism, the patient presented by Boonen et al. suffered from significant steroid myopathy [11]. Finally, Lansang et al. performed urine screens for synthetic glucocorticoids in their patient who had received epidural triamcinolone injections, and were able to detect triamcinolone in the patient's urine sixty-two days after her last injection, establishing biochemical evidence of systemic absorption [10].

The risk of iatrogenic Cushing's with HPA axis suppression in an individual patient receiving epidural steroid injections is unknown. The occurrence of this serious side effect has been reported in only a small number of cases, and a detailed review of the literature by Chou et al. found that many of the randomized placebo-controlled trials of epidural steroids did not report harm overall, and certainly not for rarer side effects [13]. Furthermore, only 5 randomized trials included more than 100 patients limiting ability to capture rare events. One could deduce that the risk for iatrogenic Cushing's is relatively low given the small number of case reports and the widespread use of epidural steroid injections, but the true incidence is unknown.

Meanwhile, the risk of adrenal suppression without overt iatrogenic Cushing's has been more clearly established. As early as 1974 [14], Burn and Langdon showed plasma cortisol levels in 50 of 56 patients to be depressed for 1-2 weeks after an epidural injection of 80 mg methylprednisolone. In 1983, Jacobs et al. [6] further studied the potential for one extradural injection of 80 mg prednisolone acetate, approximately 1 mg/kg body weight, to affect the HPA axis. After extradural drug administration, they found that patients' adrenals had a diminished ability to respond to ACTH compared to pretreatment responses despite minimally detectable drug levels in the systemic circulation. They also found evidence of central suppression in morning plasma cortisol and ACTH levels that did not resolve to pretreatment levels at 3 weeks after one injection. Similar to the Burn and Langdon study, suppression of the axis was most marked one week after the extradural steroid injection, with gradual recovery thereafter. In the case presented by Horani and Silverberg [8], the patient showed evidence of adrenal suppression over two months after her one and only epidural glucocorticoid injection. The patient was given replacement hydrocortisone 20 mg orally once daily and without dose adjustments or tapering of this dose, her HPA axis was still abnormal at eight months, but recovered by ACTH stimulation testing at twelve months.

The risk for adrenal suppression is likely higher in patients who have received serial epidural steroid injections as in our case and that of Lansang et al. [10]. This scenario was evaluated in a series by Kay et al. [15] wherein they showed that 5 of 14 patients who received 80 mg triamcinolone epidural injections weekly for 3 weeks had a subnormal response to cosyntropin one month after the last epidural injection. 

## 4. Conclusion

Hypothalamic-pituitary-adrenal axis suppression due to epidural steroid administration has been established as a potential complication of treatment. Concomitant iatrogenic Cushing's Syndrome is less common, but suggestive physical symptoms or signs should prompt referral to an endocrinologist for further testing and management. Epidural steroid injections should be held in the setting of HPA suppression even if overt Cushing's is not present. 

Given the regular use of epidural steroid treatment for chronic lumbar radicular pain, physicians administering the treatment must be aware of the potential for suppression of the HPA axis that could indicate an increased risk for adrenal crisis in the setting of stress. Some have advocated evaluation of the HPA axis via baseline cortisol levels and stimulation testing or empiric stress-dosed steroids for any patient undergoing a surgical procedure within three weeks of an epidural steroid injection [14, 15]. The case report evidence shows that some patients have suppression far beyond three weeks, supporting the use of formal testing of HPA status prior to surgery as the safest route. Conversely, it is notable that despite evidence of routine HPA suppression, there are no case reports of adrenal crisis occurring in patients with recent epidural steroid exposure. Perhaps a prudent approach would be to perform routine screening for symptoms of adrenal insufficiency in all patients who have received epidural steroids in the preceding year and to perform preoperative testing in all patients less than three weeks out from an epidural injection, those who have symptoms suspicious of adrenal insufficiency, and in those with a history of multiple injections in the preceding 4–6 months. 

## Figures and Tables

**Table 1:** Laboratory evaluation pertinent to our initial evaluation (normal values for our clinical lab in parentheses).

Time following 3rd epidural injection (time of day for lab draw)	3 wks 1505	7 wks 1005	11 wks 0838	15 wks 0830
AM serum cortisol 118.6–618 nmol/L (4.3–22.4 mcg/dL)		63.5 nmol/L (2.3 mcg/dL)	80.0 nmol/L (2.9 mcg/dL)	63.5 nmol/L (2.3 mcg/dL)

PM serum cortisol 8.53–460.8 nmol/L (3.1–16.7 mcg/dL)	16.6 nmol/L (0.6 mcg/dL)			

DHEA-S 90.2–6940 nmol/L (2.6–200 mcg/dL)		Undetectable		

Prolactin 121.7–1269.6 pmol/L (2.8–29.2 ng/mL)		208.7 pmol/L (4.8 ng/mL)		

Bioavailable testosterone 0.052–0.326 nmol/L (1.5–9.4 ng/dL)		0.045 nmol/L (1.3 ng/dL)		

Free testosterone 0.0021–0.0132 nmol/L (0.6–3.8 pg/mL)		0.0017 nmol/L (0.5 pg/mL)		

Total testosterone 0.21–0.87 nmol/L (6–25 ng/dL)		0.07 nmol/L (2 ng/dL)		

## References

[1] Armon C, Argoff CE, Samuels J, Backonja M-M (2007). Assessment: use of epidural steroid injections to treat radicular lumbosacral pain: report of the therapeutics and technology assessment subcommittee of the American Academy of Neurology. *Neurology*.

[2] Friedly J, Chan L, Deyo R (2007). Increases in lumbosacral injections in the medicare population: 1994 to 2001. *Spine*.

[3] Levin K, Hsu PS, Armon C (2009). *Lumbosacral Radiculopathy: prognosis and Treatment*.

[4] American Society of Anesthesiologists Task Force on Chronic Pain Management; American Society of Regional Anesthesia and Pain Medicine (2010). Practice guidelines for chronic pain management: an updated report by the American Society of Anesthesiologists Task Force on Chronic Pain Management and the American Society of Regional Anesthesia and Pain Medicine. *Anesthesiology*.

[5] Manchikanti L, Boswell MV, Singh V (2009). Comprehensive evidence-based guidelines for interventional techniques in the management of chronic spinal pain. *Pain Physician*.

[6] Jacobs S, Pullan PT, Potter JM, Shenfield GM (1983). Adrenal suppression following extradural steroids. *Anaesthesia*.

[7] Stambough JL, Booth RE, Rothman RH (1984). Transient hypercorticism after epidural steroid injection: a case report. *Journal of Bone and Joint Surgery. American*.

[8] Horani MH, Silverberg AB (2005). Secondary Cushing’s syndrome after a single epidural injection of a corticosteroid. *Endocrine Practice*.

[9] Tuel SM, Meythaler JM, Cross LL (1990). Cushing’s syndrome from epidural methylprednisolone. *Pain*.

[10] Lansang MC, Farmer T, Kennedy L (2009). Diagnosing the unrecognized systemic absorption of intra-articular and epidural steroid injections. *Endocrine Practice*.

[11] Boonen S, Van Distel G, Westhovens R, Dequeker J (1995). Steroid myopathy induced by epidural triamcinolone injection. *British Journal of Rheumatology*.

[12] Knight CL, Burnell JC (1980). Systemic side-effects of extradural steroids. *Anaesthesia*.

[13] Chou R, Atlas SJ, Stanos SP, Rosenquist RW (2009). Nonsurgical interventional therapies for low back pain: a review of the evidence for an American pain society clinical practice guideline. *Spine*.

[14] Burn JMB, Langdon L (1974). Duration of action of epidural methyl prednisolone. A study in patients with the lumbosciatic syndrome. *American Journal of Physical Medicine*.

[15] Kay J, Findling JW, Raff H (1994). Epidural triamcinolone suppresses the pituitary-adrenal axis in human subjects. *Anesthesia and Analgesia*.

